# Citrus sudden death-associated virus as a new expression vector for rapid *in planta* production of heterologous proteins, chimeric virions, and virus-like particles

**DOI:** 10.1016/j.btre.2022.e00739

**Published:** 2022-05-17

**Authors:** Emilyn E. Matsumura, Fei Guo, Daan Boogers, Dennis van Oevelen, Sandra T. Vu, Bryce W. Falk

**Affiliations:** a Laboratory of Virology, Wageningen University and Research, 6700 AA 8 Wageningen, the Netherlands; b Department of Plant Pathology, University of California, Davis, CA 95616, United States; c Department of Cell Biology, University of California, Davis, CA 95616, United States

**Keywords:** Citrus sudden death-associated virus, Plant virus-based expression vectors, Virus-like particles, Chimeric virions

## Abstract

The more we understand the strategies used by viruses for protein expression, the more possibilities we have to exploit viruses as expression vectors for heterologous protein production. Advances in the development of virus-based expression systems have been possible due to generation of many virus infectious clones, especially those derived from plant viruses, which have the capability for rapid and high-level transient expression of proteins in plant cells, a robust and low-cost bioreactor. In this work, we generated new replicative virus expression vectors based on a previously constructed citrus sudden death-associated virus (CSDaV) infectious cDNA clone. These vectors were generated to express the reporter green fluorescent protein (GFP) in *Nicotiana benthamiana* leaves by taking advantage of the expression strategies used by CSDaV to produce its structural proteins. We show that higher amounts of GFP can be produced from a coat protein (CP)-independent CSDaV-based vector, compared to levels of GFP expressed from a widely used non-replicative vector (pEAQ series); or GFP can be produced in fusion with the major CSDaV CP (CPp21) to be incorporated into chimeric virions. However, GFP-recombinant CSDaV virions do not appear uniformly assembled, but more likely as mosaic particles. Cryo-electron microscopy analysis from this work revealed the structures of the wild-type and the GFP-recombinant CSDaV virions, but it was not able to reveal where exactly the GFP is displayed in the chimeric virions. We show though that the incorporation of GFP-CPp21 fusion protein into virions occurs solely due to its interaction with free/non-fused CPp21, independent of other viral proteins. Therefore, individual co-expression of GFP-CPp21 and CPp21 in the same plant cells leads to the production of chimeric virus-like particles (VLPs), while GFP-CPp21 fusion protein itself is not able to self-assemble into VLPs. The new CSDaV-based expression vectors may provide an alternative platform for use in molecular farming, either for production of heterologous proteins or as scaffold for heterologous protein display.

## Highlights

• Plant viruses can be engineered as expression vectors of heterologous proteins (*hp*).
• New expression vectors were built based on citrus sudden death-associated virus (CSDaV).
• *Hp* is highly expressed when replacing the major CSDaV coat protein (CPp21).
• *Hp* can be incorporated into CSDaV (chimeric) virions when fused to the coat protein.
• Chimeric virus-like particles are produced by *in planta* co-expression of *hp*-CPp21 and CPp21.

## 1 Introduction

Since the first successful experiment expressing recombinant antibodies in plants [1], plants have become major alternative systems for production of recombinant proteins for use in a diverse range of applications, including human/animal health [2,3]. This is because plants are low-cost organisms that can be easily grown and maintained at ambient temperature, they have a very low risk of contamination with toxins or mammalian pathogens, they can be adjusted for large-scale protein production, and they can be modulated for protein N-glycosylation [4,5]. Thus, a diversity of plant-based protein expression approaches have been explored focused on stable transformation of plant nuclear or plastid genomes, or via transient expression [6,7]. Although the use of transgenic plants is the most widely used method for producing proteins in plants, the generation of transgenic plants can be time-consuming and the yield of the protein of interest is usually low, compared to the protein yields obtained from transient expression systems [8].

The plant-based transient expression, on the other hand, is achieved without the need of stable plant transformation. The expression vectors containing the protein-encoding gene of interest (usually driven by the 35S promoter derived from the cauliflower mosaic virus, CaMV) can be delivered to plant leaves either by bombardment or by *Rhizobium radiobacter* (*Agrobacterium tumefaciens*)*-*mediated (vacuum) inoculation. However, high levels of the expressed protein are only obtained for a short time and the yield is usually not sufficient for large-scale production [9,10]. Alternatively, transient expression systems using plant viruses as expression vectors have been also explored for production of several recombinant proteins. The advantages of using virus-based vectors include the rapid and the high-level expression, especially when using viruses with high replication rates [6,[8], [9], [10], [11], [12]]. Thus, the more we understand the virus strategies for protein expression, the more possibilities we have to exploit viruses as expression vectors.

Advances in the development of virus-based expression systems have been possible due to generation of many plant virus infectious clones, which can be genetically modified for gene substitution or gene insertion to produce not only individual heterologous proteins but also chimeric virus particles carrying the heterologous protein [13], [14], [15], [16], [17]. The most common and widely used virus expression vectors were engineered based on genomes of some plant RNA viruses, such as tobacco mosaic virus (TMV), cowpea mosaic virus (CMV), potato virus X (PVX) and tobacco rattle virus (TRV), as well as some DNA geminiviruses, such as bean yellow dwarf virus (BeYDV) and beet curly top virus (BCTV) [13,14,16]. However, the number of plant viruses used as expression vectors has greatly increased in the past few years [15,17,18], which has contributed to expand the possibilities for choosing the most suitable expression vector and plant host for expression of specific (recombinant) proteins.

The construction of an infectious clone derived from citrus sudden death-associated virus (CSDaV) has been previously reported [19]. CSDaV is a positive-sense, single-stranded RNA virus that belongs to the genus *Marafivirus* in the family *Tymoviridae* and has a monopartite genome of about 6.8 Kb [20]. CSDaV derived from this infectious clone locally-infects *Nicotiana benthamiana* plants via *Agrobacterium tumefaciens* inoculation and generates high amounts of viral particles after only 2 days of inoculation [19]. *In vivo* analyses of mutant versions of CSDaV revealed the strategies used by CSDaV for efficient expression of its coat proteins (CPs) [19]. In brief, CSDaV encodes for three quasi-equivalent CPs that share amino acid (aa) sequences at the C-terminus but differ from each other at the N-terminus. The major CP (CPp21) is a product of direct translation from the second start codon in the CSDaV subgenomic RNA (sgRNA), whereas the minor CPs, CPp25 and CPp23, are produced respectively by direct translation from the first start codon in the sgRNA and by proteolytic cleavage processing of the p25 precursor [19].

In this work, in order to exploit the potential usefulness of CSDaV as a vector for future translational applications, we generated new replicative vectors based on the CSDaV infectious cDNA clone [19]. The CSDaV infectious clone was modified to express the reporter green fluorescent protein (GFP) in *Nicotiana benthamiana* leaves. We show that free/non-fused GFP can be abundantly produced from a coat protein (CP)-independent CSDaV-based vector, or GFP can be produced in fusion with the major CSDaV CP (CPp21) to be incorporated into vector-derived chimeric virions. Furthermore, we demonstrate that individual co-expression of GFP-CPp21 and CPp21 proteins in same plant cells leads to the production of chimeric virus-like particles (VLPs). We envisage that results generated in this work will guide the use of these newly made virus-based vectors for future applications and offer opportunities for production of a diverse range of proteins using plant as bioreactor.

## 2 Results

### 2.1 A replicative CSDaV-based vector for *in planta* heterologous protein expression

**Fig. 1:**
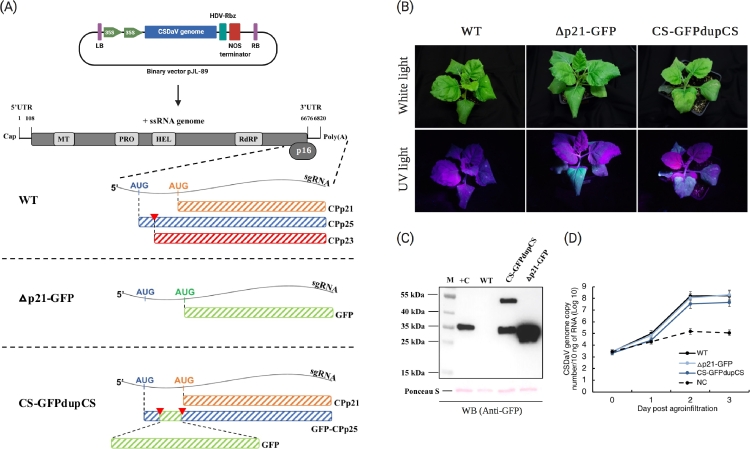
Green fluorescent protein (GFP) expression from recombinant CSDaV-based vectors. (A) Diagram showing the recombinant CSDaV-based constructs derived from a previously constructed CSDaV infectious clone (top). A schematic representation of the wild-type (WT) CSDaV genome structure is shown, from which the subgenomic RNA (sgRNA) region is enlarged to highlight the production of the three viral coat proteins: CPp21 (translated directly from the second AUG in the sgRNA); CPp25 (translated directly from the first AUG in the sgRNA); and CPp23 (proteolytically cleaved from p25 precursor) [19]. The two recombinant CSDaV-based vectors from this work (∆p21-GFP and CS-GFPdupCS) are represented by their modified sgRNA region. In ∆p21-GFP, the CPp21 coding region was replaced with that for GFP, disrupting all the CPs (since the three CPs share the same C-terminus). In CS-GFP-dupCS, the nucleotide sequence coding for the protease cleavage motif (from where CPp23 is released) was duplicated (indicated by red triangles) and the GFP coding sequence was inserted between the two cleavage motifs, leaving the CPp21 and replicase polyprotein intact as for the WT virus. The infectivity of the new recombinant CSDaV-based constructs was checked in *Nicotiana benthamiana* plants. (B) GFP fluorescence in the inoculated leaves was visualized under ultraviolet (UV) light. (C) GFP expression was confirmed by western blotting (WB) using antibody against GFP. (D) Replication of WT and the recombinant CSDaV virus genomes was assessed by a time-course RT-qPCR assay (using primers specific for the CSDaV RdRP domain). In (A): LB, T‐DNA left border; RB, T‐DNA right border; 35S, cauliflower mosaic virus 35S promoter; HDV-Rbz, hepatitis delta virus ribozyme; NOS, nopaline synthase terminator; +ss, positive-sense single-stranded; UTR, untranslated region; MT, methyltransferase; PRO, protease; HEL, helicase; RdRP, RNA-dependent RNA polymerase; p16, 16 kDa protein. In (C): M, page ruler prestained ladder; +C, positive control (GFP expressed from pEAQ vector).

The CSDaV-derived Δp21-GFP expression vector was constructed by replacing the CSDaV p21 coat protein (CPp21) coding sequence in the previously constructed CSDaV infectious clone (6082–6675 nucleotide position) [19] with the sequence coding for the reporter protein GFP (714 nucleotides; Fig. 1A). Since the full CPp21 coding sequence overlaps with the C-terminal region of the other two CSDaV CPs (CPp23 and CPp25; Fig. 1A), the lack of CPp21 in the Δp21-GFP construct also disrupts CPp23 and CPp25 and prevents the production/assembly of Δp21-GFP-derived virions. However, we hypothesized that the Δp21-GFP-derived recombinant viral genome would still be able to replicate in absence of the CPs. Thus, the goal here was to efficiently express a desired heterologous protein (GFP) by taking advantage of virus replication as well as of the strategies used by the CSDaV to highly express CPp21. Sodium dodecyl sulfate-polyacrylamide gel electrophoresis (SDS-PAGE) densitometry analysis of purified wild-type (WT)-CSDaV virions showed that > 80% of the CP subunits are composed of the CPp21, while the other < 20% are composed of the CPp23 and CPp25 together (Methods S1; Fig. S1), indicating higher expression of CPp21 over the other two CP subunits. The strategies contributing to this higher expression of CPp21 are the transcription of a CP-encoding sgRNA and the presence of a strong initiation codon that allows translation of CPp21 by ribosome leaky scanning [19] (Fig. 1A).

**Fig. 2:**
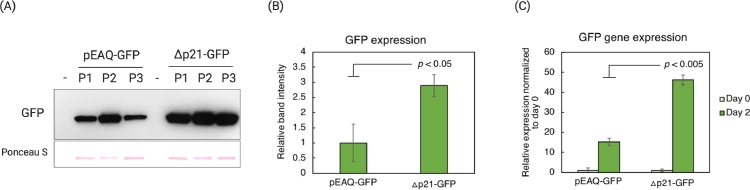
Green fluorescent protein (GFP) expression levels from a replicative CSDaV-based vector (∆p21-GFP) compared with protein expression levels from the pEAQ vector (pEAQ-GFP). (A) Western blot analysis using antibodies against GFP on total protein extracts from three independent *Nicotiana benthamiana* plants (P1, P2, P3) agroinfiltrated with either pEAQ-GFP or the CSDaV ∆p21-GFP construct; - = empty wells. (B) Intensity of protein bands in (A) were analyzed in ChemiDoc image system, and relative band intensities (to the lowest intensity value) were normalized for Ponceau S stained proteins. Bars represent average relative band intensity with standard error from three independent plants. (C) GFP expression was also compared by measuring the GFP-derived mRNA levels from *N. benthamiana* leaves agroinfiltrated with either pEAQ-GFP or ∆p21-GFP construct. Bars and error bars represent means and standard errors of three biological replicates. In (B) and (C), *p* values were calculated as two-tailed *t*-test.

The infectivity of the Δp21-GFP construct was tested in *N. benthamiana* plants via *R. radiobacter* (*A. tumefaciens*)-mediated inoculation. Under ultraviolet (UV) light illumination, GFP fluorescence was clearly visible on Δp21-GFP-infiltrated leaves at 2 days post agroinfiltration, while leaves agroinfiltrated with the WT-CSDaV infectious clone did not show any sign of fluorescence (Fig. 1B). GFP expression was confirmed by western blotting of total protein extracts obtained from the agroinfiltrated leaves (Fig. 1C). This analysis also showed much higher production of GFP derived from the Δp21-GFP vector, when compared to the amount of GFP obtained from a commonly used plant expression vector system: pEAQexpress [21] (Fig. 1C). As expected, the Δp21-GFP-derived recombinant virus was still able to replicate, accumulating similar amounts of viral RNA as the WT-CSDaV when both were inoculated in *N. benthamiana* leaves (Fig. 1D), reaching the maximum RNA accumulation at 2 days post-infiltration. Thus, GFP expressed from the ∆p21-GFP vector was quantitatively compared with GFP expressed from a pEAQ vector (pEAQ-GFP) at both protein and RNA levels (Fig. 2) on *N. benthamiana* leaves after 2 days post-agroinfiltration. At the protein level, GFP expression was detected by western blotting (Fig. 2A) and measured using a ChemiDoc image system as relative band intensities (Fig. 2B). At the RNA level, GFP-derived mRNA was relatively quantitatively analyzed by RT-qPCR (Fig. 2C). Both protein and RNA-based analyses showed that the Δp21-GFP vector can express approximately 3x higher levels of GFP in comparison to pEAQ-GFP vector (Fig. 2).

### 2.2 A modified CSDaV-based vector produces chimeric virions that incorporate CP-fused heterologous protein during virion assembly

The CSDaV-based CS-GFPdupCS vector was constructed by replacing the N-terminal first 18 aas of the CPp23 (same aas at position 13–30 in the CPp25) in the CSDaV infectious clone (6028–6081 nucleotide position) [19] with the full GFP coding sequence (Fig. 1A). This alteration disrupts the CPp23 as well as the largest CSDaV coat protein, CPp25, since the substituted 18 aas are shared by both (Fig. 1A) [19]. The full CPp21 coding sequence remained intact as in the WT-CSDaV, and the cleavage motif which is recognized by the viral protease to release the CPp23 from the p25 protein precursor [19] (Fig. 1A) was duplicated/inserted in between the GFP and CPp21 protein sequences (Fig. 1A). In this context, the normal processing of the virus replicase protein (and therefore the virus replication) is not expected to be affected. The CSDaV replicase is released from the genomic RNA-derived polyprotein by viral protease-mediated processing at the cleavage site G^1973^/S^1974^ [19], the same cleavage site encoded within the p25 precursor (translated from the sgRNA) and from where CPp23 is mainly released . This means that the alteration made to obtain the CS-GFPdupCS vector, by replacing the first 18 aas of CPp23 and leaving the first 12 aas of CPp25 intact, preserves the processing of the replicase as in the WT virus. Our goal was to obtain a recombinant CSDaV-based vector that would be able to (a) produce free heterologous protein, which would be released from a sgRNA-derived precursor by N- and C-terminal proteolytic cleavage events without affecting the processing of the virus replicase polyprotein, and (b) assemble virions, since the major CSDaV coat protein CPp21 was preserved in this construct.

**Fig. 3:**
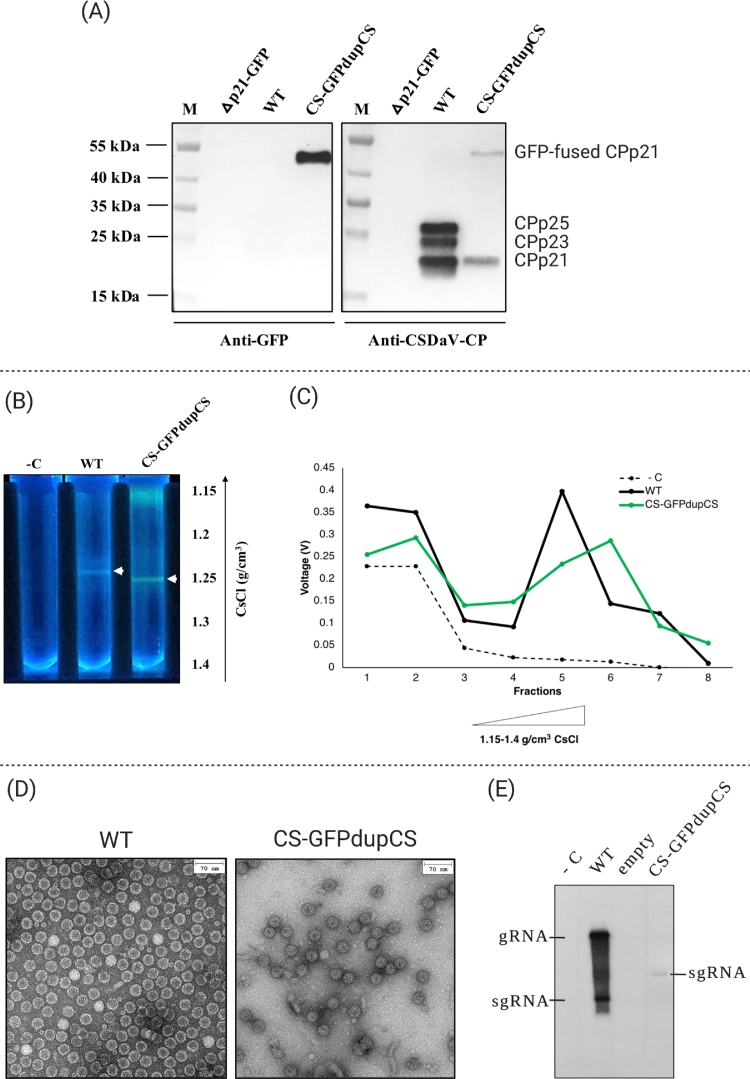
Recombinant CSDaV-based vector-derived virions can incorporate heterologous protein in the viral particles. (A) Purified virions derived from the wild-type (WT) CSDaV infectious clones, and from the new recombinant CSDaV-based vector CS-GFPdupCS were assessed for the detection of GFP and CSDaV p21 coat protein (CPp21) by western blotting using the respective anti-GFP and anti-CP antibodies. Purified sample from *Nicotiana benthamiana* leaves infiltrated with the CP-independent vector (∆p21-GFP) was also included as control. (B) WT- and recombinant CS-GFPdupCS-derived virions were subjected to cesium chloride (CsCl) gradient ultracentrifugation, white arrows indicate respective virion bands, (C) followed by fractionation while monitoring the voltage (V) value of each fraction. (D) Purified WT- and recombinant CS-GFPdupCS-derived virions were checked by transmission electron microscopy and (E) their encapsidated RNAs were verified by northern blotting (X-ray film exposed for 1 hour; result from overexposed X-ray film is shown in Fig. S2). M, page ruler prestained ladder; gRNA, genomic RNA; sgRNA, subgenomic RNA; -C, negative control.

The infectivity of the CS-GFPdupCS construct was also tested in *N. benthamiana* plants by agroinfiltration assays. Under UV light illumination, CS-GFPdupCS-infiltrated leaves showed visible GFP fluorescence at 2 days post agroinfiltration, while control plants did not show any sign of fluorescence (Fig. 1B). Western blot analysis (using antibodies against GFP) on the total protein extracts purified from the agroinfiltrated leaves detected production of both free GFP and GFP-fused CPp21 proteins (GFP-CPp21; Fig. 1C), suggesting that the viral proteolytic processing was not 100% efficient. When western blot analysis (using antibodies against both the GFP and CPp21 proteins) was performed on partially purified virions from the CS-GFPdupCS-infiltrated leaves, the free/cleaved GFP was not detected, but both fused GFP-CPp21 and free CPp21 proteins remained detectable (Fig. 3A), suggesting that the GFP-CPp21 fusion protein was incorporated into the CS-GFPdupCS-derived virions. Further purification of the WT-CSDaV- and CS-GFPdupCS-derived virions through cesium chloride (CsCl) gradient ultracentrifugation showed that virions putatively incorporating the GFP-CPp21 fusion protein banded at a density slightly greater than that of the WT virions (Fig. 3B and 3C). Transmission electron microscopy (TEM) of the purified virions confirmed the production/assembly of ∼ 30 nm icosahedral virions that resemble WT-CSDaV virions (Fig. 3D). However, northern blot analysis indicated that CS-GFPdupCS virions likely encapsidate the recombinant sgRNA (∼ 1.5 Kb) but not the recombinant genomic RNA (∼ 7.5 Kb), while WT-CSDaV virions show encapsidation of both genomic (∼ 6.8 Kb) and subgenomic RNAs (∼ 0.8 Kb; Figs. 3E and S3).

### 2.3 GFP-CPp21 fusion protein is incorporated into the chimeric CS-GFPdupCS-derived virions by interacting and co-assembling with free/non-fused CPp21

**Fig. 4:**
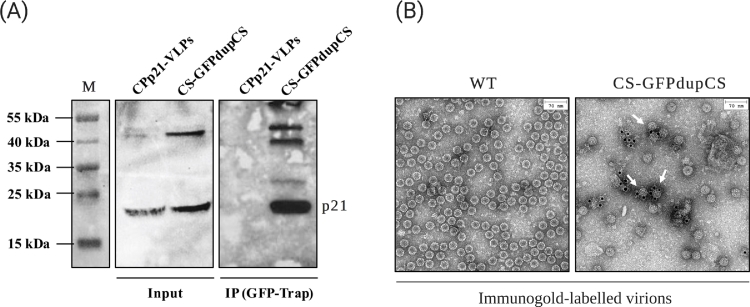
Virions derived from the recombinant CSDaV-based CS-GFPdupCS vector assemble by co-assembly interaction of the p21 coat protein (CPp21) with GFP-CPp21 fusion protein. (A) Recombinant CS-GFPdupCS-derived virions were immunopurified (IP) using GFP-Trap beads. Input and bead-bound virions were analyzed by western blotting using anti-CPp21 antibody. CPp21-derived VLPs were used as negative control. (B) Transmission electron microscopy of WT- and CS-GFPdupCS-derived virions immunogold labelled with anti-GFP antibodies (indicated with the white arrows). M, page ruler prestained ladder.

To verify co-assembly interaction between GFP-CPp21 fusion and CPp21 proteins during the process of virion assembly, CS-GFPdupCS-derived virions were immunopurified (IP) using GFP-Trap beads and then subjected to western blotting detection of the free/non-fused CPp21 protein. To ensure that CPp21 does not nonspecifically bind to the GFP-Trap beads, the full CPp21 coding sequence was cloned into pEAQexpress vector which contains the 35S promoter (Fig. S3-A) and transiently expressed on *N. benthamiana* leaves. The same protocol of crude virion preparation used to purify the WT-CSDaV and the CS-GFPdupCS-derived virions was applied using leaves agroinfiltrated with the CPp21-expressing pEAQexpress vector. The preparations were analyzed by TEM, which revealed that CPp21 can self-assemble into VLPs (Fig. S3-A). Thus, CPp21-derived VLPs were used as negative control in IP experiment. Both the input and bead-bound virions were analyzed by western blotting using antibody against the CSDaV CPp21 protein. As expected, CPp21 was detected in both input (before IP) samples: CPp21-derived VLPs and CS-GFPdupCS-derived virions (Fig. 4A). However, after GFP-Trap-mediated IP, free/non-fused CPp21 protein was only detected in immunopurified CS-GFPdupCS virions (Fig. 4A), confirming the co-assembly interaction between GFP-CPp21 fusion and CPp21 proteins.

The successful immunopurification of CS-GFPdupCS virions using GFP-trap beads also suggests that the heterologous GFP protein is likely located on the outside of the capsid surface. Immunogold labeling analysis using anti-GFP antibody followed by electron microscopy revealed the presence of gold particles surrounding the external surface of CS-GFPdupCS particles, while WT-CSDaV virions did not show any bound gold particle after same immunogold labeling procedure (Fig. 4B). However, in this experiment, only approximately 22.5% of the screened CS-GFPdupCS particles were gold-labelled (Fig. S3-B and C), indicating that the GFP-CPp21 fusion protein may not be abundantly incorporated into all of the particles and/or the GFP is not always displayed on the outside of the particles’ surface.

### 2.4 Cryo-EM analysis reveals almost indistinguishable structures between WT and CS-GFPdupCS-derived CSDaV virions

**Fig. 5:**
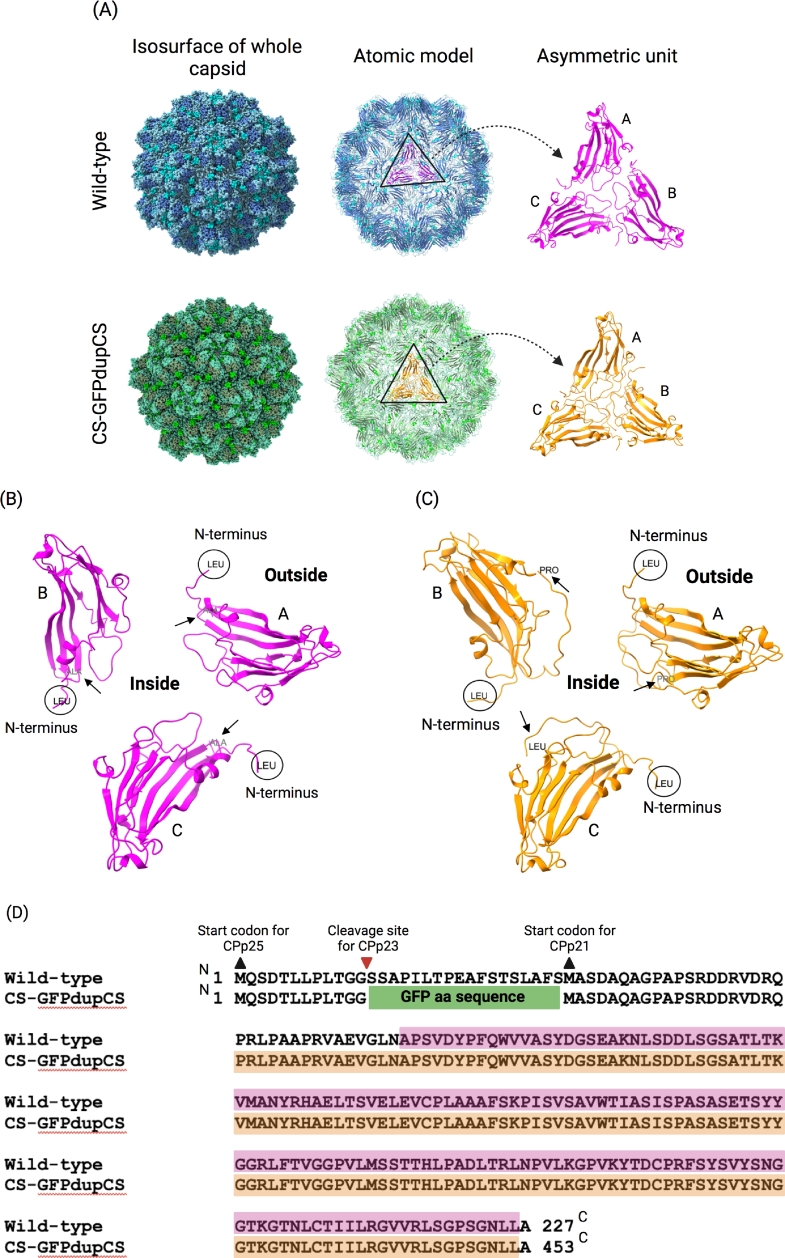
Cryo-EM structures of wild-type (WT) and GFP-CSDaV (CS-GFPdupCS) virions. (A) A panel showing the cryo-EM maps of whole virus capsid (left), the atomic model of the shell component (middle), and a single asymmetric unit (right) of the WT- and GFP-CSDaV (top and bottom, respectively). (B) and (C) Enlarged view of a single asymmetric unit in their positions on the WT- and GFP-CSDaV capsids, respectively. The asymmetric unit is composed of three quasi-equivalent chains (A, B and C). ‘Inside’ and ‘Outside’ respectively indicates the inside and outside surface of the capsid. The N-terminal region of each chain is circled and indicated, and the C-terminal region is pointed with a black arrow. (D) Sequence alignment of the coat protein domain of the WT- and GFP-CSDaV indicating where the GFP coding sequence was inserted (not to scale) in the CS-GFPdupCS virus and which amino acid residues were visible in the cryo-EM density maps and included in the models constructed for the WT-CSDaV (highlighted in pink) and for the GFP-CSDaV (highlighted in orange). ^N^ indicates the N-terminal region; ^C^ indicates C-terminal region.

In attempts to reveal where (if in the inside or outside) GFP is displayed on the GFP-recombinant CSDaV virions (CS-GFPdupCS), we have determined the cryo-EM structures of both the WT- and the CS-GFPdupCS-derived virions. In total, 175,869 and 12,732 particles were used for structure reconstruction of WT and CS-GFPdupCS virions, respectively, yielding density maps at a resolution of 3.1 Å for WT and 3.4 Å for CS-GFPdupCS virus (Fig. 5; see Table S2 for refinement statistics). The whole capsid models of the two types of particles are almost indistinguishable (Fig. 5A) and reveals a *T* = 3 icosahedral symmetry that gives particles with a diameter of about 30 nm. The primary sequence of the CSDaV major CP (CPp21) was used to generate initial models (by automated homology modeling) for the WT and CS-GFPdupCS virions. Both final models were built as a single asymmetric unit that comprises three chains (A, B, and C; Fig. 5). Each subunit of the asymmetric unit has typical Jelly Roll folds which consist of eight antiparallel beta sheets. These three subunits have triangular arrangement with quasi-3-fold symmetry (Fig. 5A). Analysis comparing our results with protein domain structure annotations available in the CATH database [22], revealed that the CSDaV capsid has similar subunit arrangement and tertiary structure with desmodium yellow mottle tymovirus (DYMV) [23], in which chain A forms pentameric capsomeres while chains B and C form hexametric capsomeres. However, a large N-terminal region of the CPs was not visible in the electron density map, and therefore these residues are not shown in the final atomic model. Residues 1−65 were not visible for the CSDaV WT CPs, while residues 1−276 (including the full aa sequence of the GFP) were not visible for the GFP-fused CP (Fig. 5B and 5C, respectively). In case the N-terminus of CSDaV CPs projects outwards from the virions, as suggested by the GFP Trap-mediate IP and by the immunogold labeling experiments (Fig. 4), the missing densities for this region would not be surprising, due likely to a high flexibility of outside virion surface domains [3,24]. However, analysis on the position of each asymmetric unit chain in the capsid of WT-CSDaV particles indicates that the N-terminal region of the CPs is displayed towards the inside of the particle, whereas the C-terminal region is directed to the outside surface of the particle. This might indicate that the N-terminal region, non-visible in the electron density map, could potentially be hidden inside of the particle. On the other hand, a larger domain from the N-terminus of the CPp21 is visible for the CS-GFPdupCS virions, in comparison to the visible domain observed for the WT-CSDaV virions (Fig. 5D). Intriguingly, in chains B and C from the asymmetric unit built for the CS-GFPdupCS, it is possible to observe that both the N- and C-terminal regions are directed towards the outside of the particle (Fig. 5C). Thus, it is indeed possible that the GFP incorporated into the CS-GFPdupCS-derived virions, by fusion to the N-terminus of the CPp21, is displayed on the outside surface of the particles but it is not visible in the cryo-EM map due to the high flexibility of this domain. However, cryo-EM structure analysis could not confirm this hypothesis and the possibility of N-terminus CP displays on the inside of the capsid (hidden in the cryo-EM map) cannot be discarded.

### 2.5 Interactions of transiently co-expressed GFP-CPp21 fusion and CPp21 proteins produce chimeric virus-like particles

**Fig. 6:**
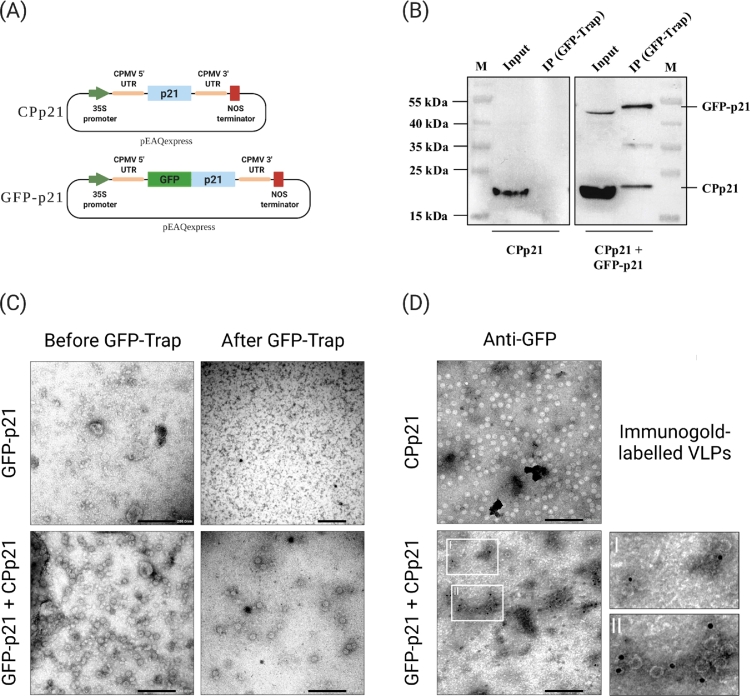
Co-assembly interaction between CSDaV p21 coat protein (CPp21) and GFP-fused CPp21 protein co-expressed in *Nicotiana benthamiana* leaves produces chimeric virus-like particles (VLPs). (A) Schematic representation (not to scale) of the pEAQexpress‐derived plasmids used in the transient expression experiment. (B) *N. benthamiana* leaves co-transiently expressing CPp21 and GFP-fused CPp21 proteins were used for VLPs purification. Purified VLPs were further immunopurified (IP) using GFP-Trap beads. Input and bead-bound VLPs were analyzed by western blotting using anti-CPp21 antibody. CPp21-derived VLPs were used as negative control. (C) Transmission electron micrographs of GFP-Trap purified GFP-p21 fusion protein and GFP-Trap purified (CPp21 + GFP-fused CPp21)-derived VLPs. (D) Transmission electron micrographs of CPp21- and (CPp21 + GFP-fused CPp21)-derived VLPs immunogold labelled with anti-GFP antibody. The two regions selected with white squares, and identified as I and II, are enlarged for better visualization of the gold particles. Black bars in 6C and 6D represent 200 nm.

Since our results demonstrated that CS-GFPdupCS-derived virions are assembled by co-assembly interactions between GFP-CPp21 fusion and CPp21 proteins, we assessed whether these proteins also interact and co-assemble, leading to the production of chimeric VLPs when they are co-transiently expressed in plants, independently of the virus replication or other viral proteins. This was assessed by performing co-agroinfiltration of pEAQexpress vectors individually containing the coding sequences of the GFP-CPp21 fusion and CPp21 proteins (Fig. 6A) on *N. benthamiana* plants. Two days post agroinfiltration, GFP-CPp21 fusion/CPp21 co-infiltrated leaves were harvested for VLPs purification. Purified VLPs were subjected to GFP-Trap-mediated IP, followed by western blotting detection of free/non-fused CPp21 protein. CPp21-derived VLPs were used as control.

Similar to results obtained from the previous co-IP experiment on the CS-GFPdupCS-derived virions, free/non-fused CPp21 protein was detected in both GFP-CPp21 fusion/CPp21- and CPp21-derived VLPs when the inputs (before IP) were analyzed (Fig. 6B), while after GFP-Trap-mediated IP, free/non-fused CPp21 protein was only detected in VLPs purified from the GFP-CPp21 fusion/CPp21 co-infiltrated leaves (Fig. 6B). These results further confirm the co-assembly interaction of GFP-CPp21 fusion and CPp21 proteins and indicate that this interaction does not rely on the presence of other CSDaV proteins. Additionally, the VLPs immunopurified from the GFP-CPp21 fusion/CPp21 co-infiltrated leaves were examined in the TEM, which confirmed the assembly of VLPs that resemble the CS-GFPdupCS-derived virions (Fig. 6C - bottom). The same VLPs purification method was applied to *N. benthamiana* leaves transiently expressing GFP-CPp21 fusion protein only, but no VLPs were detected by TEM, which indicates that the fusion protein cannot self-assemble into VLPs and its incorporation occurs solely due to its co-assembly interaction with free/non-fused CPp21 (Fig. 6C - top). Immunogold labeling analysis using anti-GFP antibody also revealed the presence of gold particles surrounding the external surface of GFP-CPp21 fusion/CPp21-derived VLPs, while CPp21-derived VLPs did not show any bound gold particles after same immunogold labeling procedure (Fig. 6D).

## 3 Discussion

The generation of many plant virus infectious clones during the past decades has expanded our knowledge about the strategies used by viruses for protein expression, enabling the exploitation of many of these viruses as vectors for heterologous protein production [17]. In our previous study, we constructed an infectious cDNA clone of CSDaV, a plant marafivirus, which efficiently locally infects *N. benthamiana* plants via *A. tumefaciens* inoculation [19]. In this same previous work, *in vivo* analyses of mutant versions of CSDaV infectious clone revealed the expression strategies/mechanisms used by CSDaV for efficient replication and high expression of its structural proteins [19]. These results not only demonstrated that the CSDaV infectious cDNA clone is stable and tolerates genetic modifications but also indicates the potential of using this virus as a vector for translational applications. Here, we described the construction of two CSDaV-derived vectors for *in planta* heterologous protein expression. These vectors were generated based either on a full virus strategy (containing the entire virus genome) or on a gene substitution strategy (by replacing the gene encoding for one of the virus proteins) to express GFP as the reporter heterologous protein. Our approach was to insert the GFP-coding sequence within the CSDaV sgRNA region which is highly transcribed from an internal viral promoter (termed as marafibox) and from which the viral coat proteins are produced at high levels [19].

The high expression levels obtained for the major CSDaV coat protein (CPp21), when considering the WT-CSDaV infectious clone, does not only rely on the transcription of the coat proteins-encoding sgRNA but also on the presence of a strong context initiation codon that specifically allows high CPp21 translation by ribosome leaky scanning [19]. Replacing the CPp21 gene for that encoding GFP in the CSDaV infectious clone culminated with the generation of a CP-independent replicative virus vector (Δp21-GFP) that gave expression of approximately 3x higher levels of free/non-fused GFP, compared to levels of GFP expressed from a widely used non-replicative vector from the pEAQ series [21]. This quantitative analysis was performed on Δp21-GFP-agroinfiltrated *N. benthamiana* leaves at 2 days post agroinfiltration, demonstrating that this replicative vector can generate high levels of heterologous protein within a very short time frame. Thus, we envisage that the CSDaV-derived Δp21-GFP vector has the potential to be translated to the applications of large-scale protein production using plants as bioreactors.

Besides the expression of free/non-fused heterologous proteins, we also demonstrated that the CSDaV infectious clone can be modified to incorporate heterologous protein into assembled virion capsids, just as it has been demonstrated for other plant virus-based vectors [25,26]. This was demonstrated by fusing the GFP coding sequence to that for CPp21 in the full-length CSDaV infectious clone to generate the construct CS-GFPdupCS, referring to the fact that the GFP-coding sequence was inserted between two identical protease cleavage sites (CS) in frame with CPp21. We showed that this new infectious clone-derived recombinant CSDaV is able to replicate and assemble into particles that resemble the WT-CSDaV virions. Additionally, the GFP gene insert was retained during replication of the new recombinant virus, which is an important result as many of replicating virus vectors have the tendency of deleting heterologous inserted sequences. However, the increase in genome size with the insertion of the GFP gene resulted in lack of encapsidation of the recombinant CSDaV genomic RNA in the CS-GFPdupCS-derived particles. A previous work has demonstrated that CPp21 from *Maize rayado fino virus* (MRFV), which is also a marafivirus, can self-assemble into VLPs, but CP mRNA encapsidation only occurs when both MRFV CP subunits (CPp21 and CPp25) are present during particle assembly [27]. Since two (CPp25 and CPp23) of the three CSDaV CP subunits are disrupted with the fusion of the GFP to the CPp21 in the CS-GFPdupCS construct, it is possible that the absence of CPp25 and CPp23 may also affect the encapsidation of the genomic RNA, although the increase in genome size seems more likely to be the main reason, since we showed that the recombinant sgRNA was still being encapsidated by the CS-GFPdupCS-derived particles. Thus, further vector improvement, such as by removing CSDaV coding sequences that are not essential for viral replication, virion assembly and RNA encapsidation, is needed in order to reduce the size of the recombinant virus genome, and therefore increase the probability of virus genome encapsidation and production of complete recombinant virions (if that is the aim).

Furthermore, analysis of the CS-GFPdupCS-derived virions suggested self-assembly of the CSDaV CPp21 (without the need of neither CPp23 or CPp25) and production of chimeric particles by the incorporation of the GFP-CPp21 fusion protein during particle assembly. Additional experiments confirmed that CPp21 is also able to self-assemble into VLPs, but this ability is disrupted with the fusion of GFP, likely due to steric hindrance. This could be a drawback of using CPp21-derived VLPs platform for making chimeric VLPs, as VLP platforms derived from other plant viruses, such as grapevine fanleaf virus (GFLV), for example, showed that GFLV CP-GFP fusion protein is still able to efficiently assemble into VLPs [28]. The incorporation of GFP-CPp21 fusion protein into chimeric VLPs or virions occurs solely due to its interaction with free/non-fused CPp21, independent of other viral proteins. However, the chimeric VLPs or virions do not seem to be uniformly assembled, and co-expression of CPp21 with the GFP-CPp21 fusion protein likely produce mosaic particles, similar to what has been reported for turnip crinkle virus (TCV)-derived mosaic VLPs [29]. Our analysis suggests that only less than 25% of the CS-GFPdupCS-derived virions displayed detectable GFP on the outside surface of the particle. The other 75% of assembled virions either do not incorporate the GFP-CPp21 fusion protein or the GFP is probably hidden inside of the particle. Cryo-EM structure analysis could not reveal where the GFP is displayed on the CS-GFPdupCS virions because a large N-terminal region of the GFP-CPp21 fusion protein, including the full GFP aa sequence was not visible in the electron density map. Similarly, densities of the first 65 N-terminal residues of the WT-CSDaV CPs were not visible, making the solved WT- and CS-GFPdupCS-CSDaV capsid structures almost indistinguishable. Additionally, analysis on the position of each asymmetric unit chain in the capsids of WT- and CS-GFPdupCS-virions was inconclusive in showing whether the N-termini of the CSDaV CPs (where the GFP is inserted) displays towards the outside or inside of the virion capsids. Our observations indicate that it is indeed possible that the GFP incorporated into the CS-GFPdupCS virions is displayed on the outside surface of the particles, but it could not be detected in the cryo-EM map due to the high flexibility of this domain (outside surface and flexible N-terminal domain hypothesis). However, the possibility that the N-terminus of the CP is displayed on the inside of the capsid cannot be discarded (inside surface and hidden N-terminal domain hypothesis).

Although the CSDaV vector systems generated in this work will likely need to be optimized for production of specific heterologous proteins and/or for other translational applications, we have demonstrated that CSDaV-based vectors are versatile, they can be modified to either express free/non-fused heterologous protein or to incorporate heterologous protein into assembled chimeric virions or VLPs (at least when applied to the marker protein GFP). This new virus-based tool could be attractive for production of other heterologous proteins, possibly including human/animal proteins, and complement the list of well-known plant viral-based expression vectors that have been successfully used in molecular farming, such as TMV [30], CPMV [31], and PVX [32] and in which plants are used as relatively inexpensive bioreactors, when compared for instance with animal cell-based systems.

## 4 Material and methods

### 4.1 Construction of the viral vectors and other plasmids

The new CSDaV-derived vectors as well as the additional plasmids from this work were constructed using the NEBuilder HiFi DNA Assembly Cloning system (NEB, Ipswich, MA, USA), following the manufacturer's instructions. The set of primers (Table S1) used in the construction of each plasmid was designed using the SnapGene software version 3.3 (http://www.snapgene.com/). The vectors and insert fragments were obtained by PCR with their respective primers using CloneAmp HiFi PCR premix (Clontech), following the manufacturer's protocol. The amplicons were gel purified using Zymoclean™ Gel DNA Recovery Kit (Zymo Research Corporation, Irvine, CA, USA) and the HiFi DNA Assembly reactions were performed by combining 100 ng of the vector, 100 ng of the respective fragment and 1x of HiFi DNA Assembly premix. The reactions were incubated at 50 °C for 15 min and then transformed into *Escherichia coli* (DH5α) competent cells. Plasmids were purified from the transformed colonies using the QIAprep Spin Miniprep Kit (Qiagen, Valentia, CA, USA) and confirmed by Sanger sequencing using primers designed to cover the insert region (Table S1).

### 4.2 Agroinfiltration assays

The agroinfiltration assays were performed as described in Matsumura et al. [19]. Briefly, the confirmed plasmids were introduced into *A. tumefaciens* (strain GV3101) by electroporation method. Pre-inoculum cultures of the transformed agrobacteria were used to inoculate l-MESA medium [LB medium supplemented with 10 mM 2-(N-morpholino) ethanesulfonic acid (MES) and 20 μM acetosyringone] containing 50 μg/mL of kanamycin and 10 μg/mL of rifampicin and grown overnight at 28 °C to until reach to an OD600 nm of 0.8–1.2. The cells were pelleted for 10 min at 5500 g, resuspended in agroinfiltration solution (10 mM MgCl2, 100 μM acetosyringone and 10 mM MES, pH 5.7) to an OD600 nm of 0.8 and incubated at room temperature (RT) for 3 h in the dark. Agroinfiltrations were performed on the abaxial surface of 4 expanded leaves of 3-weeks *N. benthamiana* plants. Plants infiltrated with *A. tumefaciens* containing the WT CSDaV infectious clone or a mutated and non-replicative full-length CSDaV clone were used as controls. The agroinfiltrated plants were maintained in a greenhouse under constant conditions and daily monitored until GFP fluorescence was observed (under UV light) .

### 4.3 Real time RT-qPCR

Virus replication was checked by absolute quantification of the viral RNA accumulated over time (at 0, 1, 2 and 3 days post infiltration, dpi). GFP-derived mRNA accumulation was measured by relative quantification. In both RT-qPCR analysis, total RNAs were obtained from agroinfiltrated leaves using Trizol reagent (Life Technologies, Carlsbad, CA, USA), following the manufacturer's instructions. Total RNA samples were treated with RQ1 RNase-free DNase I (Promega, Madison, WI, USA) and purified with phenol:chloroform. cDNAs were synthesized by reverse transcription reactions using the High-Capacity cDNA Reverse Transcription Kit (Applied Biosystems, Carlsbad, CA, USA), following the manufacturer's protocol. qPCR was performed to detect either the CP gene of CSDaV or the GFP gene using 5 μL of SsoAdvanced Universal SYBR Green Supermix (Bio-Rad, Foster City, CA, USA), 300 nM of each of the respective primers (Table S1), 1 μL of the 1:5 diluted cDNA and water up to a total volume of 10 μL. qPCR reactions were subjected to 95 °C for 3 min, followed by 40 cycles of 95 °C for 10 s and 55 °C for 30 s. For the absolute quantification analysis, same procedure was performed with serial diluted samples of a plasmid containing the full-length cDNA genome of the CSDaV. The plasmid copy number for each dilution was calculated as described in Plumet and Gerlier [33] to generate a standard curve which was used to estimate the viral RNA copy number in the tested samples. For relative quantification analysis, GFP-derived mRNA levels were normalized to protein phosphatase 2A (PP2A) endogenous *N. benthamiana* gene and calculated using Pfaffl's method [34]. The statistical difference between the test and the control was calculated by *t*-test (*p* < 0.05). All RT-qPCR experiments were performed in three biological replicates and three technical repeats.

### 4.4 Protein and virion/VLP purification and protein detection

Total proteins were extracted from 100 mg of plant material (agroinfiltrated leaves) in 300 μL of protein extraction buffer (100 mM Tris–HCl pH 7.5, 100 mM EDTA pH 8.0, 5 mM DDT, 150 mM NaCl and 0.1% v/v Triton X-100). Both virions and VLPs were purified from agroinfiltrated leaves at 2 dpi in 0.2 M of sodium acetate buffer (pH 5.0) and precipitated with 8% (w/v) polyethylene glycol (PEG8000) and 1% (w/v) NaCl, as described in Matsumura et al. [19]. Further virions purification was performed by loading the crude-purified virions on a cesium chloride (CsCl) gradient (from bottom to top: 1.40, 1.30, 1.25, 1.20 and 1.15 g/cm^3^ of CsCl in 10 mM Tris–HCl buffer pH 7.8) followed by an ultracentrifugation of 4 h at 50.000 rpm and 11 °C (Beckman SW65 Ti Rotor). Fractions containing the virions were either detected at 260 nm using a density gradient fractionator (model 185, ISCO) or extracted from the tube using an 18-gage needle syringe. Fractions with virions were diluted in 9 mL of 10 mM Tris–HCl buffer pH 7.8 and centrifuged for 45 min at 45.000 rpm (Beckman 70.1 Ti Rotor). Pellets were suspended in the same buffer.

Protein extracts and virions/VLPs were separated by SDS-PAGE using precast 12.5% polyacrylamide gels (Bio-Rad, Hercules, CA, USA) and transferred to nitrocellulose membranes for immunodetection of either the CPp21 CSDaV protein (using antibody against the C-GPAPSRDDRVDRQP peptide) or GFP (#PA1–980A, Invitrogen). The goat anti-rabbit IgG-HRP conjugate (Bio-Rad, Hercules, CA, USA) was used as secondary antibody and proteins were detected by using the SuperSignal West Pico Chemiluminescent Substrate (Thermo Fisher Scientific) and ChemiDoc Touch Imaging System (Bio-Rad, Hercules, CA, USA). GFP relative expression levels were assessed by measuring the intensity of GFP bands, which were normalized for Ponceau S-stained proteins, and relatively compared to band intensities of a control sample (the lowest intensity value).

### 4.5 TEM and northern blot analysis

The purified virions and VLPs were loaded onto Formvar-carbon-coated grids, stained with 1% uranyl acetate and examined with a JEOL 2100F transmission electron microscope (Peabody, MA, USA; available at the UC Davis BioEM facility) at an accelerating voltage of 200 kV.

To check whether the GFP-recombinant CSDaV-derived virions encapsidate the recombinant viral RNAs, RNAs extracted from the purified virions were denatured with glyoxal at 55 °C for 30 min, electrophoresed on 1% agarose gels and transferred to Hybond-NX membrane (GE Amersham, Piscataway, NJ, USA). Membrane was subjected to UV-cross-linking, followed by staining with methylene blue. ^32^P-labelled probe was made based on the nucleotide sequences of the CPp21, as described in Matsumura et al. [19], to detect both the genomic and subgenomic CSDaV positive-strand RNAs. Membranes were hybridized with the ^32^P-labelled probe in Ultra-hyb Buffer (Ambion) at 42 °C overnight. After hybridization, the membrane was washed once in 2 × SSC (1 × SSC is 0.15 m NaCl plus 0.015 m sodium citrate) added of 0.1% SDS (RT for 15 min), once in 0.5 × SSC/0.1% SDS (RT for 15 min) and once with 0.1 × SSC/0.1% SDS (65 °C for 15 min). Detection of RNA bands was performed by exposing the membrane to Premium X-Ray film (Phenix Research Products).

### 4.6 Co-immunopurification

GFP-recombinant virions or VLPs were immunopurified using an anti-GFP single domain antibody conjugated to agarose beads (GFP-Trap; Chromotek). Briefly, 300 μL of purified virions/VLPs were added to 25 μL of equilibrated bead slurry and mixed/rotate end-over-end for 1 hour at 4 °C. Virions/VLPs bound to the GFP-Trap agarose beads were separated from the unbound proteins through Pierce Spin Columns (Thermo Fisher, Rockford, IL, USA), following the manufacturer's instructions. Bound virions/VLPs were eluted from the beads by adding 50 μL of 0.2 M glycine (pH 2.5), incubating it for 10 min at RT followed by centrifugation at 2.500 rpm for 2 min. The eluted samples were neutralized with 5 μl of 1 M Tris base (pH 10.4) and directly used for western blotting for detection of the CSDaV CPp21 protein as described above.

### 4.7 Immunogold labeling TEM

GFP-recombinant purified virions or VLPs (5 μL) were loaded onto formvar/carbon coated nickel grids (TED PELLA, # 01800N-F) and incubated for 5 min at RT in a moist chamber. Grids were then blocked in a drop of blocking buffer (1% BSA, 10 mM TrisHCl pH 7.4, 100 mM NaCl and 0.1% tween 20) for 10 min and incubated in a drop of anti-GFP antibody (1:200 dilution) for 1 hour at RT. Grids were washed 5x with TE buffer and incubated for 10 min in blocking solution, followed by incubation on a drop of 1:30 diluted secondary antibody conjugated with gold beads (GAR-gold 10 nm; TED PELLA #15,726) for 1 hour at RT. Grids were washed 5x with TE buffer, and stained with 1% uranyl acetate solution for examination with the electron microscope.

### 4.8 Cryo-electron microscopy

An aliquot (3 μl) of purified virions (WT or GFP-conjugated) was applied to a glow-discharged holey carbon grid (300 mesh Quantifoil 1.2/1.3) for plunge freezing in liquid nitrogen using FEI Vitrobot™ Mark III (8 s with −2 mm off set). Cryo-EM data was acquired at 200 kV on a Thermofisher Glacios electron microscope equipped with a Gatan K3 direct electron detector, available at the UC Davis BioEM Core Facility. Micrographs were recorded at 56,818x (0.88 Å/pixel) magnification using K3 super-resolution mode (0.44 Å/pixel before binning) and dose fractionation (75 frames, 0.8 e/Å2 per frame). Parallel beam illumination and coma-free alignment was applied using SerialEM [35]. A total of 2481 and 7898 micrographs were collected for the WT and GFP-recombinant virus, respectively.

Movie stacks were motion corrected and dose weighted using Motioncor2 [36]. Data were imported in cisTEM [37] for CTF determination, particle picking, 2-D classification and 3-D reconstruction of initial model. These data were imported in Relion 3.1 [38] for automated high resolution 3-D reconstruction. The final resolution was evaluated using Fourier shell correlation (threshold=0.143 criterion). The datasets and refinement statistics are summarized in Table S2.

The first CP atomic model was built solely based on the A subunit of the Cryo-EM density map of the WT-CSDaV virions at 5-fold symmetry. Residue positions were manually adjusted, and the peptide chain of the major CSDaV CP (CPp21) was created and docked to other two subunits in the asymmetric unit. Subunits were manually adjusted to accommodate the non-equivalent conformations and to better fit the densities. After docking the *ab initio* model into the whole EM density map, the asymmetric unit density map was segmented out using UCSF Chimera [39]. Subsequent model refinement was performed with iterative manual adjustment in *Coot* [40], and automatic real space refinement/validation by Phenix [41]. For GFP-recombinant CSDaV model, the WT was used as the template for map segmentation and initial refinement, and the iterative real space refinement/validation were performed using Phenix and *Coot*.

Cryo-EM data are publicly available wwPDB at accession codes 7SQY and 7SQZ, and EMDB-IDs EMD-25,397 and EMD-25,398.

## Author contributions

EEM and BWF conceived and designed research; EEM performed most of the experiments; FG, DB, DvO, and STV performed part of the experiments; EEM, FG and BWF analyzed the data; EEM and BWF wrote the manuscript. All authors read and approved the manuscript.

## Declarations of Competing Interest

The authors have no conflicts of interest to declare.

## Appendix Supplementary materials

Image, application 1

## References

[1] Hiatt A., Cafferkey R., Bowdish K. (1989). Production of antibodies in transgenic plants. Nature.

[2] Tiwari S. (2009). Plants as bioreactors for the production of vaccine antigens. Biotechnol. Adv..

[3] Marsian J. (2017). Plant-made polio type 3 stabilized VLPs-a candidate synthetic polio vaccine. Nat. Commun..

[4] Strasser R. (2008). Generation of glyco-engineered Nicotiana benthamiana for the production of monoclonal antibodies with a homogeneous human-like N-glycan structure. Plant Biotechnol. J..

[5] Lomonossoff G.P., D'Auost M.A (2016). Plant-produced biopharmaceuticals: a case of technical developments driving clinical deployment. Science.

[6] Naseri Z. (2019). Virus-based vectors: a new approach for production of recombinant proteins. J. Appl. Biotechnol. Rep..

[7] Schillberg S., Finnern R. (2021). Plant molecular farming for the production of valuable proteins - critical evaluation of achievements and future challenges. J. Plant Physiol..

[8] Yamamoto T. (2018). Improvement of the transient expression system for production of recombinant proteins in plants. Sci. Rep..

[9] Gleba Y., Klimyuk V., Marillonnet S. (2005). Magnifection–a new platform for expressing recombinant vaccines in plants. Vaccine.

[10] Desai P.N., Shrivastava N., Padh H. (2010). Production of heterologous proteins in plants: strategies for optimal expression. Biotechnol. Adv..

[11] Yamamoto T. (2018). Improvement of the transient expression system for production of recombinant proteins in plants. Sci. Rep..

[12] Wang A., Wang A., Ma S. (2012). Molecular Farming in Plants: Recent Advances and Future Prospects.

[13] Salazar-Gonzalez J.A., Banuelos-Hernandez B., Rosales-Mendoza S. (2015). Current status of viral expression systems in plants and perspectives for oral vaccines development. Plant Mol. Biol..

[14] Hefferon K. (2017). Plant virus expression vectors: a powerhouse for global health. Biomedicines.

[15] Lico C., Chen Q., Santi L. (2008). Viral vectors for production of recombinant proteins in plants. J. Cell Physiol..

[16] Ruiz-Ramon F. (2019). Second generation of pepino mosaic virus vectors: improved stability in tomato and a wide range of reporter genes. Plant Methods.

[17] Abrahamian P., Hammond R.W., Hammond J. (2020). Plant virus-derived vectors: applications in agricultural and medical biotechnology. Annu. Rev. Virol..

[18] Peyret H., Lomonossoff G.P. (2015). When plant virology met Agrobacterium: the rise of the deconstructed clones. Plant Biotechnol. J..

[19] Matsumura E.E. (2019). Rescue of Citrus sudden death-associated virus in Nicotiana benthamiana plants from cloned cDNA: insights into mechanisms of expression of the three capsid proteins. Mol. Plant Pathol..

[20] Maccheroni W. (2005). Identification and genomic characterization of a new virus (Tymoviridae family) associated with citrus sudden death disease. J. Virol..

[21] Sainsbury F., Thuenemann E.C., Lomonossoff G.P. (2009). pEAQ: versatile expression vectors for easy and quick transient expression of heterologous proteins in plants. Plant Biotechnol. J..

[22] Orengo C.A., Michie A.D., Jones S., Jones D.T., Swindells M.B., Thornton J.M. (1997). CATH–a hierarchic classification of protein domain structures. Structure.

[23] Larson S.B. (2000). Refined structure of desmodium yellow mottle tymovirus at 2.7 A resolution. J. Mol. Biol..

[24] Grinzato A. (2020). Atomic structure of potato virus X, the prototype of the Alphaflexiviridae family. Nat. Chem. Biol..

[25] Hamamoto H.S., Nakagawa N., Hashlda E., Matsunaga Y., Takemoto S., Watanabe Y.O. (1993). A new tobacco mosaic virus vector and its use for the systemic production of angiotensin-1-converting enzyme inhibitor in transgenic tobacco and tomato. Biotechnology.

[26] Sugiyama Y.H., H. Takemot S., Watanabe Y., Okada Y. (1995). Systemic production of foreign peptides on the particle surface of tobacco mosaic virus. FEBS Lett..

[27] Hammond R.W., Hammond J. (2010). Maize rayado fino virus capsid proteins assemble into virus-like particles in Escherichia coli. Virus Res..

[28] Belval L. (2016). Display of whole proteins on inner and outer surfaces of grapevine fanleaf virus-like particles. Plant Biotechnol. J..

[29] Castells-Graells, R.L.G.P.S.K., *Production of Mosaic Turnip Crinkle Virus-Like Particles Derived By Coinfiltration of Wild-Type and Modified Forms of Virus Coat Protein in Plants.*, in *Virus-Derived Nanoparticles For Advanced Technologies.*, L.G. Wege C., Editor. 2018, Humana Press: New York, NY. 10.1007/978-1-4939-7808-3_1 29869231

[30] Lindbo J.A. (2007). TRBO: a high-efficiency tobacco mosaic virus RNA-based overexpression vector. Plant Physiol..

[31] Gopinath K. (2000). Engineering cowpea mosaic virus RNA-2 into a vector to express heterologous proteins in plants. Virology.

[32] Komarova T.V. (2006). New viral vector for efficient production of target proteins in plants. Biochemistry (Mosc).

[33] Plumet S., Gerlier D. (2005). Optimized SYBR green real-time PCR assay to quantify the absolute copy number of measles virus RNAs using gene specific primers. J. Virol. Methods.

[34] Pfaffl M.W. (2001). A new mathematical model for relative quantification in real-time RT-PCR. Nucleic Acids Res..

[35] Schorb M. (2019). Software tools for automated transmission electron microscopy. Nat. Methods.

[36] Zheng S.Q. (2017). MotionCor2: anisotropic correction of beam-induced motion for improved cryo-electron microscopy. Nat. Methods.

[37] (2018). cisTEM software for cryo-EM. Nat. Methods.

[38] Zivanov J. (2018). New tools for automated high-resolution cryo-EM structure determination in RELION-3. Elife.

[39] Pettersen E.F. (2004). UCSF Chimera–a visualization system for exploratory research and analysis. J. Comput. Chem..

[40] Emsley P. (2010). Features and development of Coot. Acta Crystallogr. D Biol. Crystallogr..

[41] Liebschner D. (2019). Macromolecular structure determination using X-rays, neutrons and electrons: recent developments in Phenix. Acta Crystallogr. D Struct. Biol..

