# Supplementary material for: Citrus sudden death-associated virus as a new expression vector for rapid in planta production of heterologous proteins, chimeric virions, and virus-like particles
Source: Biotechnol Rep (Amst). 2022 May 17;35:e00739. doi: 10.1016/j.btre.2022.e00739 (PMC9130518; doi:10.1016/j.btre.2022.e00739)
Supplement: Supplementary file 1 [file mmc1.docx]

1. Supporting experimental procedures

**Methods S1:** *Densitometry analysis on the amount of each coat protein*

Purified wild-type CSDaV virions were diluted from 1x to 0.8, 0.6 and 0.4x and analyzed by SDS-PAGE for densitometry analysis. Briefly, virions samples were mixed with 2x protein loading buffer (100 mM Tris-HCl pH 6.8, 200 mM DTT, 4% SDS, 20% glycerol, 0.2% bromophenol blue and 5% β-mercaptoethanol) and boiled for 5 min at 95 °C. Samples were loaded on a precast 12.5% polyacrylamide gel (Bio-Rad, Hercules, CA, USA) and electrophoresis was performed at 150V for about 40 min. The gel was then stained with Coomassie Brilliant Blue R-250 (Fisher Biotech, Fair Lawn, NJ, USA) and scanned using a ChemiDoc Touch Imaging System (Bio-Rad, Hercules, CA, USA). Intensities of the individual protein bands (CPp25, CPp23 and CPp21) were analyzed/quantified in a ChemiDoc image system by background baseline subtraction and expressed as relative percentages of lane (Figure S1).

2. Supporting Figures


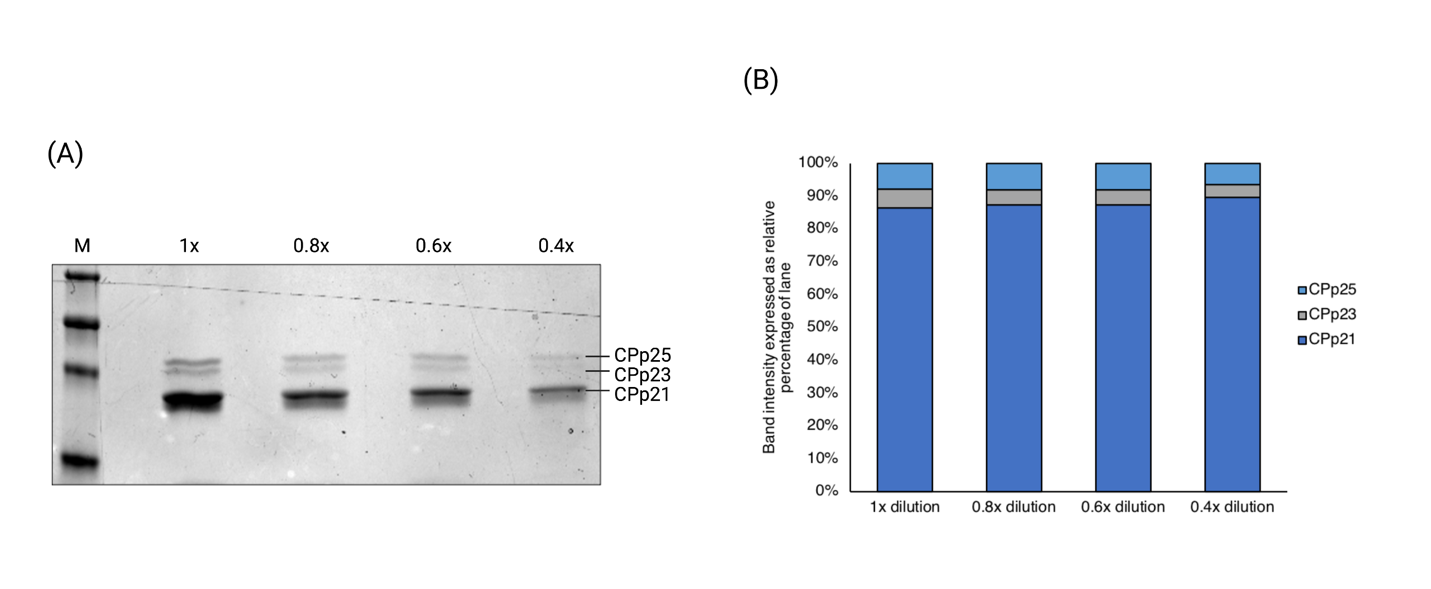


**Figure S1**: Densitometry analysis on the amount of each coat protein (CP) subunit present in the wild-type (WT)-CSDaV capsid. (A) SDS-PAGE of purified WT-CSDaV virions at 1x, 0.8x, 0.6x and 0.4x dilutions showing visually differences in CP subunits composition. M, page ruler prestained ladder. (B) Intensity of protein bands in (A) were analyzed in ChemiDoc image system, and band intensities were expressed as percentages of lane.


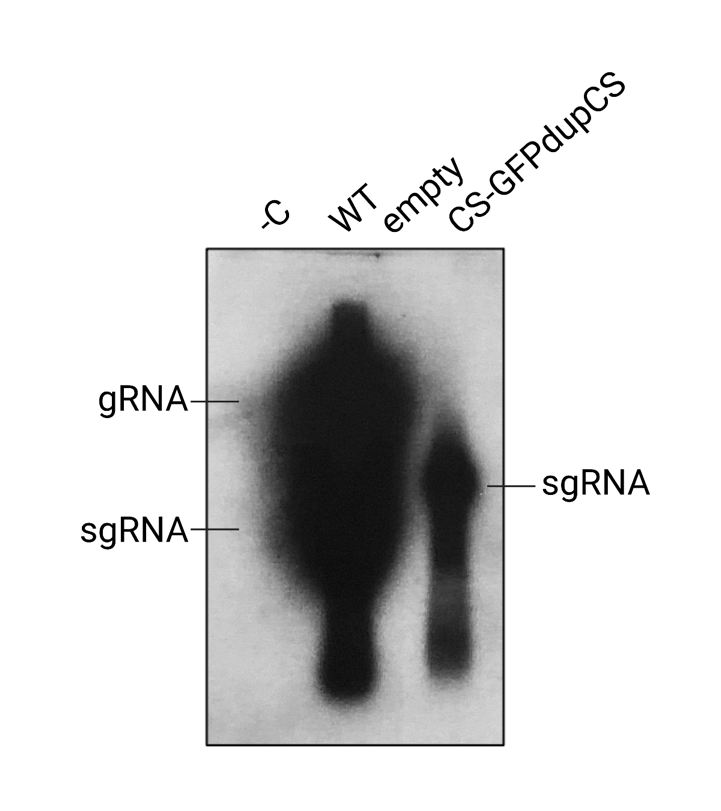


**Figure S2:** Northern blotting of RNAs obtained from wild-type (WT)-CSDaV- and recombinant CS-GFPdupCS-derived virions. The presented blot (same blot from Figure 3E) shows the result from X-ray film exposed for 24 hours. gRNA, genomic RNA; sgRNA, subgenomic RNA; -C, negative control.


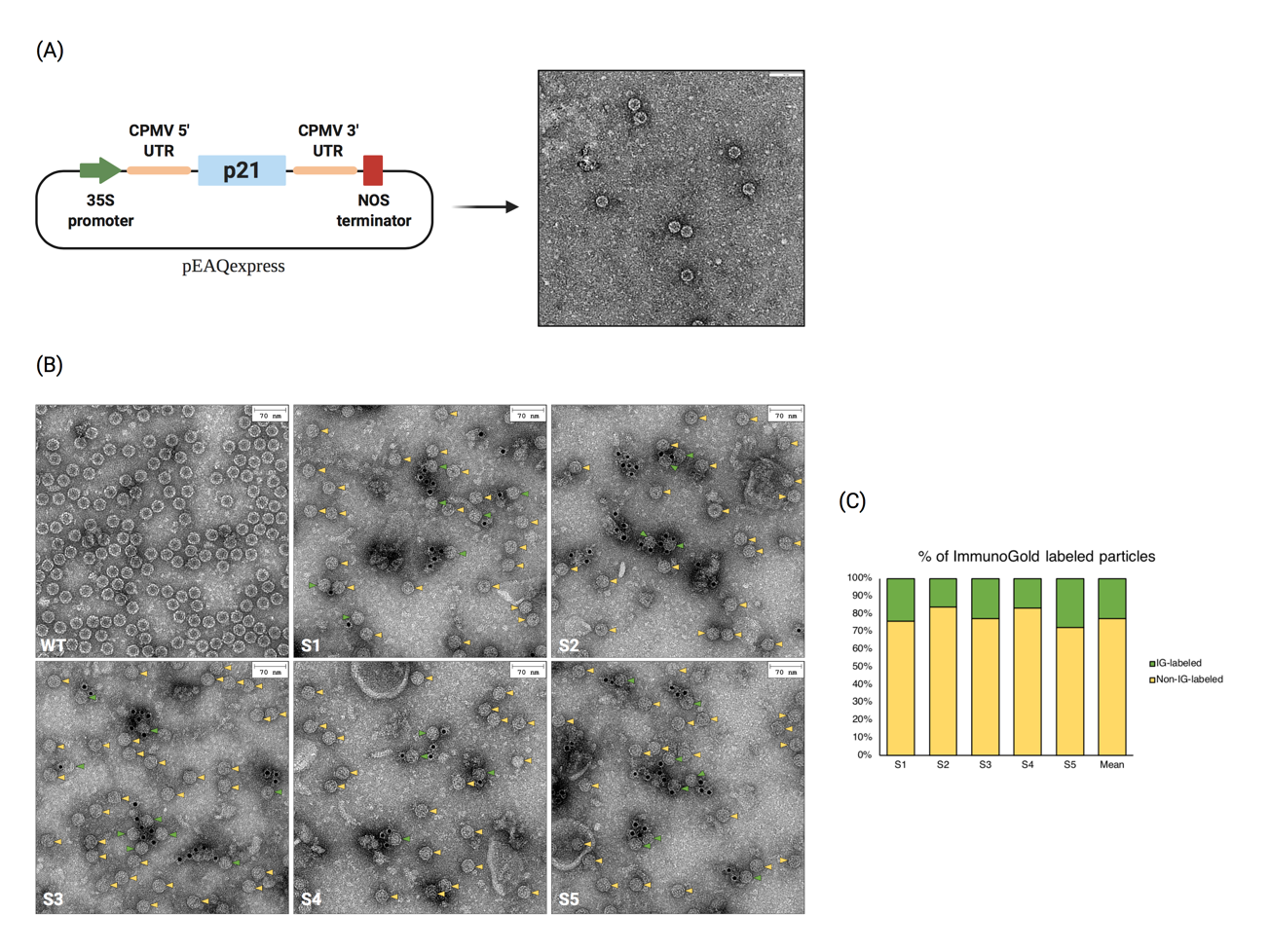


**Figure S3**: Self-assembly of the CSDaV CPp21 was verified by transient expression in *Nicotiana benthamiana* leaves followed by virus-like particles (VLPs) purification. (A) Schematic representation of the pEAQexpress‐derived plasmid constructed to express CPp21 (left) and transmission electron micrographs of purified CPp21-derived VLPs (right). 35S promoter, cauliflower mosaic virus 35S promoter; NOS, nopaline synthase terminator; CPMV, Cowpea mosaic virus; UTR, untranslated region. Immunogold labeling of recombinant CS-GFPdupCS-derived virions with anti-GFP antibody allowed for the estimation of the percentage of particles likely displaying the GFP domain on the outside surface. (B) Transmission electron microscopy of wild-type CSDaV- (WT) and CS-GFPdupCS-derived virions (represented by five different grid spots: S1-S5) immunogold labelled with anti-GFP antibodies. Yellow triangles, non-labeled particles. Green triangles, gold-labeled particles. (C) Bar graph expressing the percentage of immunogold-labeled particles based on the representative micrographs showed in (B). The last bar represents the mean value of S1, S2, S3, S4 and S5.

3. Supporting tables

**Table S1**: Primers used in this study. The purpose of each primer is presented.

| **Primer** | **Sequence (5'-3')** | **Purpose** |
| --- | --- | --- |
| Δp21-GFP_FragF | CCTCGCTTTCTCCATGGTGAGCAAGGGCGAGGAG | Δp21-GFP cloning  (fragment) |
| Δp21-GFP_FragR | CCTGAAGAGGCTCGCTTACTTGTACAGCTCGTCCATGCC |  |
| Δp21-GFP_VecF | GCGAGCCTCTTCAGGTGAAG | Δp21-GFP cloning  (Vector) |
| Δp21-GFP_VecR | CATGGAGAAAGCGAGGGAGGTG |  |
| CS-GFPdupCS_FragF | TGGTGGTGTGAGCAAGGGCGAGGAGC | GFPdupCS cloning  (Fragment) |
| CS-GFPdupCS_FragR | ACCAGTGAGCTTGTACAGCTCGTCCATGCC |  |
| CS-GFPdupCS_VecF | TGTACAAGCTCACTGGTGGTTTCTCCATGG | GFPdupCS cloning  (Vector) |
| CS-GFPdupCS_VecR | TTGCTCACACCACCAGTGAGAGGCAG |  |
| Mbox_F | GGTGAATTGCTTCAGCACTTTCAAGTACCATTCAT | Sequencing |
| 3UTR_R | CTAAGCGAGAAGATTACCGGAGGG |  |
| CPp21_FragF | CCAAATTCGCGAATGATGGCCAGCGATGCC | CPp21-pEAQ cloning  (Fragment) |
| CPp21_FragR | TAAAGGCCTCGACTACTAAGCGAGAAGATTACCGGAGGG |  |
| GFP-CPp21_FragF | GCGAATGGTGAGCAAGGGCGAGGAG | GFP-CPp21-pEAQ cloning  (Fragment) |
| GFP-CPp21_FragR | TCGCTGGCCATGGAGAAACC |  |
| pEAQ-F | TAGTCGAGGCCTTTAACTCTGGT | pEAQ clonings  (Vector) |
| CPp21_pEAQ-R | CATTCGCGAATTTGGGCAGAATATACAG |  |
| GFP-CPp21_pEAQ-R | TTGCTCACCATTCGCGAATTTGGGCAG |  |
| CSDaV-CP-qPCR_F | TCTTGCTGCAGCCTTCTCCA | qPCR |
| CSDaV-CP-qPCR_R | ACAGGACCGCCAACAGTGAA |  |
| CSDaV-CPF | GCCATCTACACCACACTCTC | Make probe for Northern blot |
| CSDaV-CPR | TTGGAGTAGACGGAGTAGGA |  |

**Table S2**: Cryo-EM data collection, reconstruction, model refinement and validation statistics.

|  | **WT-CSDaV** | **GFP-CSDaV ( CS-GFPdupCS)** |
| --- | --- | --- |
| **Data Collection and processing** |  |  |
| Microscope | Glacios | Glacios |
| Detector | Gatan K3 | Gatan K3 |
| Magnification | 56,818 | 56,818 |
| Voltage (kV) | 200 | 200 |
| Electron dose (e/Å^2^) | 60 | 60 |
| Pixel size (Å) | 0.88 | 0.88 |
| Software | SerialEM | SerialEM |
| **Reconstruction** |  |  |
| Software | cisTEM / Relion | Relion |
| Number of particles | 175,869 | 12,732 |
| Box size (pixels) | 512 | 512 |
| Final resolution (Å) | 3.1 | 3.4 |
| EMDB ID | 25398 | 25397 |
| **Model** |  |  |
| Software | Phenix / *Coot* | Phenix / *Coot* |
| PDB ID | 7SQZ | 7SQY |
| Map CC (around atoms) | 0.9 | 0.89 |
| Model composition |  |  |
| Non-hydrogen atoms | 3582 | 3860 |
| Protein residues | 482 | 522 |
| Number of chains | 3 | 3 |
| RMS deviations |  |  |
| Bond lengths (Å) | 0.005 | 0.003 |
| Bond angles (°) | 0.649 | 0.578 |
| Ramachandran Plot |  |  |
| Favored (%) | 95.6 | 95.9 |
| Allowed (%) | 4.4 | 4.1 |
| Disallowed (%) | 0 | 0 |
| MolProbity score | 1.84 | 1.75 |
| Clash score | 10.2 | 8.67 |
| Rotamer outliers (%) | 0 | 0 |
